# Supplementary material for: Inclusive fitness forces of selection in an age-structured population
Source: Commun Biol. 2023 Sep 5;6:909. doi: 10.1038/s42003-023-05260-9 (PMC10480192; doi:10.1038/s42003-023-05260-9)
Supplement: Supplementary file 2 — Supplementary Information [file 42003_2023_5260_MOESM2_ESM.pdf]

Supplementary Information for

Inclusive fitness forces of selection in an age-structured population

Mark Roper[1,2*], Jonathan P. Green[1], Roberto Salguero-Gómez[1,3], Michael B. Bonsall[1].

Author affiliations:

1. *Department of Biology, University of Oxford, 11a Mansfield Road, Oxford, OX1 3SZ, UK.*
2. *Institute for Biodiversity and Ecosystem Dynamics, University of Amsterdam, Amsterdam, The Netherlands.*
3. *Max Planck Institute for Demographic Research, Konrad-Zuse-Straße 1, 18057 Rostock, Germany*

Corresponding author: Mark Roper
Email: markroper67@gmail.com

This PDF file includes:

Appendix C: Supplementary methods for numerical applications
Appendix D: Further derivations for dw_{1x}
Supplementary Figures 1-4

Appendix C: Supplementary methods for numerical applications

The following represents a general method for modelling arbitrarily complex social transfers between (st)age classes, that was used to create Figures 2 and 3 in the main text, but could be populated from real population data. To populate the elements of the inclusive fitness matrix (***W***), we first model the propensity of each age class to contribute to the survival and reproduction of each age class, including its own. These genetic offspring transfer propensities can be conceptualised as two different matrices, one for survival and one for reproduction. The entry $\mathbf{T}_{yx}$ represents the relative propensity for age class x to contribute to the survival or reproduction of age class y. If $\mathbf{T}_{yx} = 0$, an individual in age class x has no contribution to the survival or reproduction of an individual in age class y. Allowing for flexibility in the direction of transfers, we allow these propensities to take the functional form of

$$\mathbf{T}_{yx} = T(x, y, C_x, C_y)$$

[C1]

Here, C represents the stage of each age class, which could be, for example, pre-reproductive (juveniles), mature reproductive, mature non-reproductive, or post-reproductive. The form of **[C1]** allows for the relative propensity to be a function of the age and stage of both actor and recipient. For simplicity in the examples we provide (see **Main Text**), we only focus on propensities that vary by the age classes of actor and recipient. For our population with post-reproductive survival (Fig. 2) we input a 1 into each element of the **T** matrix for survival in which the column represents a post-reproductive age class and the row represents a juvenile age class. This means that every post-reproductive age class has the same relative propensity to contribute to the survival of each juvenile age class. Every other element in **T** (survival) is set to 0, and every element in **T** (reproduction) is 0. On the contrary, for our population with pre-reproductive help (Fig. 3), we input a 1 into each element of the **T** matrix for reproduction with a column representing a pre-reproductive age class and a column representing a reproductive age class. This means every pre-reproductive age class has the same relative propensity to contribute to the reproduction of each reproductive age class. Every other element in **T** (reproduction) = 0, and every element in **T** (survival) = 0.

The next step is to model the proportions of age-specific survival and reproduction that are due to the social environment. The total social transfers received by age class x is $\sum_z T^z_{x+1,x}$ for survival at age x and $\sum_z T^z_{1,x}$ for reproduction at age class x, which are taken as absolute proportions of the background demographic rates $p(x)$ and $b(x)$. These proportions may vary by age and stage, and different age classes within the same stage could have different proportions stripped from their survival and reproduction. For example, very young juveniles might owe their survival more to other members of the group than older juveniles. Here, again for simplicity, we model cases were 10, 20 and 30% of juvenile survival (Fig.1; Supplementary Figure 1; Supplementary Figure 2) or 10, 20 and 30% of adult reproduction is due to the social environment. These contributions are independent of age within the stage classification *i.e.* the same proportion is stripped from all juvenile or adult stage classes within the same iteration of the model.

We can then quantify indirect fitness contributions by distributing the stripped genetic offspring equivalents that are the result of the social environment. First, a focal individual aged x is responsible for a fraction of the total genetic offspring contributions to the survival of age class z from all age classes (*i.e.* $\frac{T^{x}_{z+1,z}}{\sum_y T^{y}_{z+1,z}}$). The fraction that is distributed to age class x is then:

$$T^{x}_{z+1,z} = (N-1)s_z t^{x}_{z+1,z}\hat{r}(x)$$

[C2]

where $(N-1)s_z$ is the expected number of individuals alive on the patch aged z with the focal individual aged x, $\hat{r}(x)$ is the relatedness of a focal individual aged x to a random breeder on the patch, and $t^{x}_{z+1,z}$ represents the fraction of the total social contributions to the survival of age class z that are due to age class x, and can be written more explicitly as

$$t^{x}_{z+1,z} = \sum_j T^{j}_{z+1,z}\frac{s_x \mathbf{T}_{zx}}{\sum_j s_j \mathbf{T}_{zj}}$$

[C3]

The same logic applies to reproductive contributions. For each age class, these indirect genetic contributions are compiled into the inclusive fitness matrix $\boldsymbol{W}$, along with the direct components of survival ($\dot{p}(x)$) and reproduction ($\dot{b}(x)$). The inclusive fitness forces of selection acting on a mutant allele that alters the rate of survival between age x and $x+1$ or rate of reproduction at age x can then be calculated according to **[8]** and **[10]** in the main text. Code for the numerical solutions explored in the main text is available in a separate file.

Appendix D: Further derivations for dw_{1x}

The inclusive fitness effect of a mutant allele that causes a change in the direct rate of reproduction of a focal individual aged age *x* on it's direct fitness can be displayed as

$$\frac{dw_{1x}(1)}{d\dot{b}(x)} = \dot{b}(x)[(1-d)g(x) + (1-c)d\bar{g}] - \dot{b}'(x)[(1-d)g'(x) + (1-c)d\bar{g}]$$

[D1]

The following working displays the simplification of **[D1]:**

$$\frac{dw_{1x}(1)}{d\dot{b}(x)} = \dot{b}(x)(1-d)g(x) + \dot{b}(x)(1-c)d\bar{g} - \dot{b}'(x)(1-d)g'(x) - \dot{b}'(x)(1-c)d\bar{g}\,,$$

$$\frac{dw_{1x}(1)}{d\dot{b}(x)} = \dot{b}(x)(1-d)g(x) - \dot{b}'(x)(1-d)g'(x) + (1-c)d\bar{g}\left[\dot{b}(x) - \dot{b}'(x)\right],$$

$$\frac{dw_{1x}(1)}{d\dot{b}(x)} = (1-d)\left[\frac{\dot{b}(x)[(1-p(x)+(N-1)\bar{p}]}{b(x)(1-d)+(N-1)\bar{b}(1-d)+N\bar{b}(1-c)d} - \frac{\dot{b}'(x)[(1-p(x)+(N-1)\bar{p}]}{b'(x)(1-d)+(N-1)\bar{b}(1-d)+N\bar{b}(1-c)d}\right] + (1-c)d\bar{g}[b(x)-b'(x)]$$

The terms inside the brackets are manipulated using algebraic subtraction and, after simplification, results in:

$$\frac{dw_{1x}(1)}{d\dot{b}(x)} = (1-d)\left[\frac{[b(x)-b'(x)]\,[(1-p(x)+(N-1)\bar{p}]\left[(N-1)\bar{b}(1-d)+N\bar{b}(1-c)d\right]}{\left[b(x)(1-d)+(N-1)\bar{b}(1-d)+N\bar{b}(1-c)d\right]\left[b'(x)(1-d)+(N-1)\bar{b}(1-d)+N\bar{b}(1-c)d\right]}\right] + (1-c)d\bar{g}[b(x)-b'(x)]$$

Further simplification results in the expression:

$$\frac{dw_{1x}(1)}{d\dot{b}(x)} = (1-d)[b(x)-b'(x)]\left[\frac{[(1-p(x)+(N-1)\bar{p}]\left[(N-1)\bar{b}(1-d)+N\bar{b}(1-c)d\right]}{\left[b(x)(1-d)+(N-1)\bar{b}(1-d)+N\bar{b}(1-c)d\right]\left[b'(x)(1-d)+(N-1)\bar{b}(1-d)+N\bar{b}(1-c)d\right]}\right] + (1-c)d\bar{g}[b(x)-b'(x)]$$

which using definitions in **Methods Appendix B** is equal to:

$$\frac{dw_{1x}(1)}{d\dot{b}(x)} = (1-d)[b(x)-b'(x)][g(x)(1-h(x)] + (1-c)d\bar{g}[b(x)-b'(x)]$$

which can finally be simplified to:

$$\frac{dw_{1x}(1)}{d\dot{b}(x)} = d\dot{b}(x)[(1-d)g(x)(1-h(x)) + (1-c)d\bar{g}]$$

[D2]

The inclusive fitness effect of a mutant allele that causes a change in the direct rate of reproduction of a focal individual aged age *x* on the fates of offspring it indirectly produced through the reproduction of others can be displayed as

$$\frac{dw_{1x}(2)}{d\dot{b}(x)} = \sum_z T^x_{1,z}\,(1-d)g(x) - \sum_z T^x_{1,z}\,(1-d)g'(x)$$

[D3]

The following working displays the simplification of **[D3]:**

$$\frac{dw_{1x}(2)}{d\dot{b}(x)} = \sum_z T^x_{1,z}\,(1-d)\left[\frac{[(1-p(x)+(N-1)\overline{p}]}{b(x)(1-d)+(N-1)\overline{b}(1-d)+\ N\overline{b}(1-c)d} - \frac{[(1-p(x)+(N-1)\overline{p}]}{b'(x)(1-d)+\ (N-1)\overline{b}(1-d)+\ N\overline{b}(1-c)d}\right]$$

The terms inside the brackets are manipulated using algebraic subtraction and, after simplification, results in:

$$\frac{dw_{1x}(2)}{d\dot{b}(x)} = \sum_z T^x_{1,z}\,(1-d)\left[\frac{[b(x)-b'(x)]\ [(1-p(x)+(N-1)\overline{p}](1-d)}{\left[b(x)(1-d)+(N-1)\overline{b}(1-d)+\ N\overline{b}(1-c)d\right]\left[b'(x)(1-d)+\ (N-1)\overline{b}(1-d)+\ N\overline{b}(1-c)d\right]}\right]$$

Further simplification and using definitions in **Methods Appendix B** results in the expression:

$$\frac{dw_{1x}(2)}{d\dot{b}(x)} = \ d\dot{b}(x)(1-d)\left[\frac{[(1-p(x)+(N-1)\overline{p}]\left[\sum_z T^x_{1,z}\,(1-d)\right]}{\left[b(x)(1-d)+(N-1)\overline{b}(1-d)+\ N\overline{b}(1-c)d\right]\left[b'(x)(1-d)+\ (N-1)\overline{b}(1-d)+\ N\overline{b}(1-c)d\right]}\right]$$

which can finally be simplified to:

$$\frac{dw_{1x}(2)}{d\dot{b}(x)} = -\,d\dot{b}(x)\left[(1-d)g(x)\dot{I}(x)\right]$$

[4]

Finally, the inclusive fitness effect of a mutant allele that causes a change in the direct rate of reproduction of a focal individual aged age *x* on the fates of other offspring born on the patch can be written as

$$\frac{dw_{1x}(3)}{d\dot{b}(x)} = (N-1)\bar{F}(1-d)g(x) - (N-1)\bar{F}(1-d)g'(x)$$

[D5]

The following working displays the simplification of **[D5]:**

$$\frac{dw_{1x}(3)}{d\dot{b}(x)} = (N-1)\bar{F}(1-d)\left[\frac{[(1-p(x)+(N-1)\bar{p}]}{b(x)(1-d)+(N-1)\bar{b}(1-d)+N\bar{b}(1-c)d} - \frac{[(1-p(x)+(N-1)\bar{p}]}{b'(x)(1-d)+(N-1)\bar{b}(1-d)+N\bar{b}(1-c)d}\right]$$

The terms inside the brackets are manipulated using algebraic subtraction and, after simplification, results in:

$$\frac{dw_{1x}(3)}{d\dot{b}(3)} = (N-1)\bar{F}(1-d)\left[\frac{[b(x)-b'(x)]\,[(1-p(x)+(N-1)\bar{p}](1-d)}{[b(x)(1-d)+(N-1)\bar{b}(1-d)+N\bar{b}(1-c)d][b'(x)(1-d)+(N-1)\bar{b}(1-d)+N\bar{b}(1-c)d]}\right]$$

Further simplification and using definitions in **Appendix B** results in the expression:

$$\frac{dw_{1x}(3)}{d\dot{b}(x)} = d\dot{b}(x)(1-d)\left[\frac{[(1-p(x)+(N-1)\bar{p}][(N-1)\bar{F}(1-d)]}{[b(x)(1-d)+(N-1)\bar{b}(1-d)+N\bar{b}(1-c)d][b'(x)(1-d)+(N-1)\bar{b}(1-d)+N\bar{b}(1-c)d]}\right]$$

which can finally be simplified to:

$$\frac{dw_{1x}(3)}{d\dot{b}(x)} = -\,d\dot{b}(x)[(1-d)g(x)\dot{k}(x)]$$

[D6]

These offspring are related to the focal individual by $\hat{r}(x)$. Equations **[D2]**, **[D4]**, and **[D6]** can then be summed to give **[B10]** in **Appendix B**.

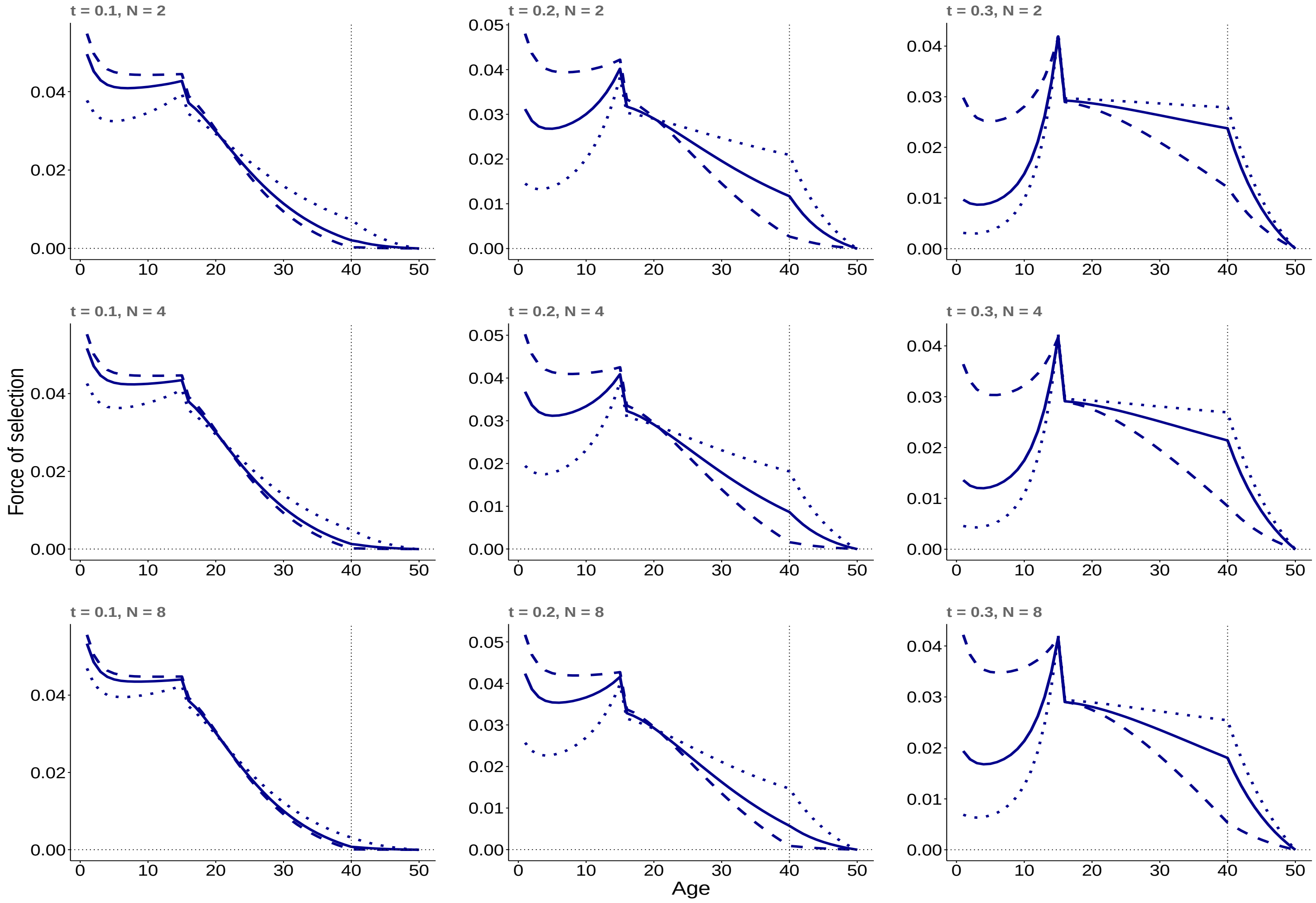

Supplementary Figure 1. The force of selection acting on survival rate at age x in a population with post-reproductive survival (**see Fig. 2**) as a function of *(i)* the magnitude of the social contribution from post-reproductive individuals to the survival of juveniles, where t = the fraction of juvenile survival that is due to post-reproductive individuals, *(ii)* the number of individuals on the patch (N) and *(iii)* the juvenile dispersal rate in the population: 0.2 (dotted line), 0.5 (continuous line), and 0.8 (broken line). The vertical dotted line at age 40 represents the age at which reproduction ceases.

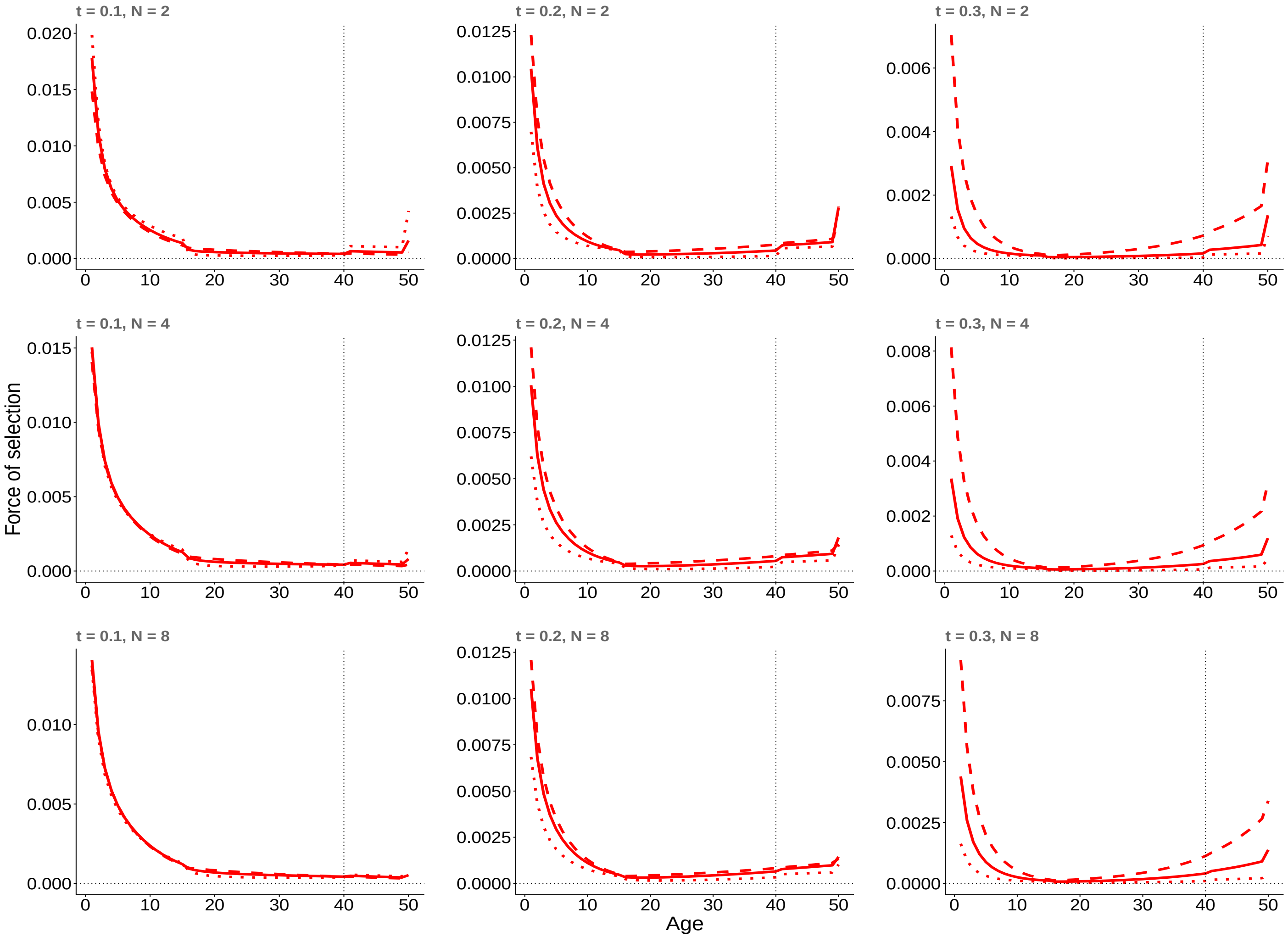

Supplementary Figure 2. The force of selection acting on reproduction at age x in a population with post-reproductive survival (**see Fig. 2**) as a function of *(i)* the magnitude of the social contribution from post-reproductive individuals to the survival of juveniles, where t = the fraction of juvenile survival that is due to post-reproductive individuals, *(ii)* the number of individuals on the patch (N) and *(iii)* the juvenile dispersal rate in the population: 0.2 (dotted line), 0.5 (continuous line), and 0.8 (broken line). The vertical dotted line at age 40 represents the age at which reproduction ceases.

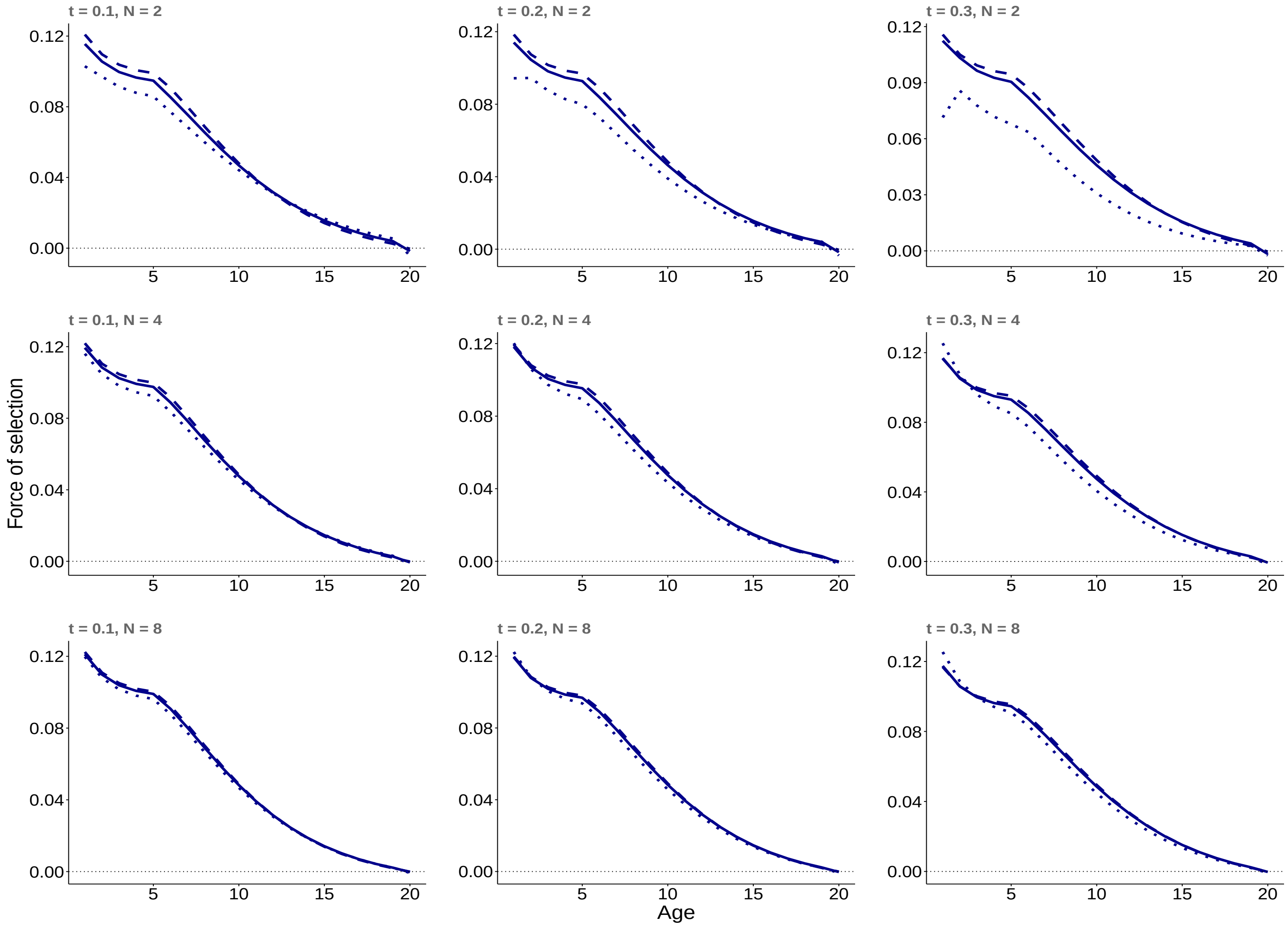

Supplementary Figure 3. The force of selection acting on survival at age x in a population with pre-reproductive helpers (**see Fig. 3**) as a function of *(i)* the magnitude of the social contribution from pre-reproductive individuals to the reproduction of reproductive-aged adults, where t = the fraction of reproduction that is due to pre-reproductive individuals, *(ii)* the number of individuals on the patch (N) and *(iii)* the juvenile dispersal rate in the population: 0.2 (dotted line), 0.5 (continuous line), and 0.8 (broken line).

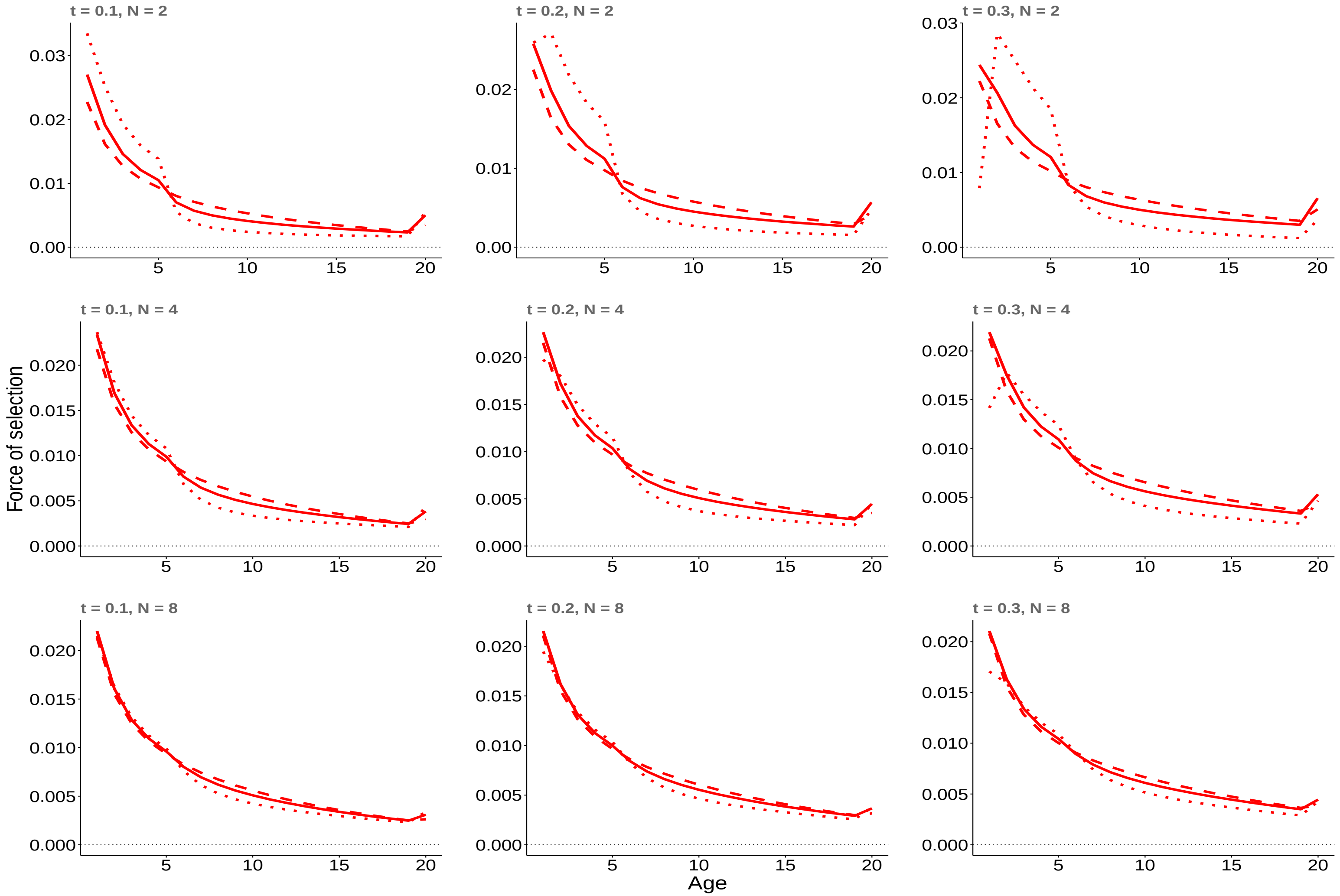

Supplementary Figure 4. The force of selection acting on reproduction at age x in a population with pre-reproductive helpers (**see Fig. 3**) as a function of *(i)* the magnitude of the social contributions from pre-reproductive individuals to the reproduction of reproductive-aged adults, where t = the fraction of reproduction that is due to pre-reproductive individuals, *(ii)* the number of individuals on the patch (N) and *(iii)* the juvenile dispersal rate in the population: 0.2 (dotted line), 0.5 (continuous line), and 0.8 (broken line).
