# Supplementary material for: Inclusive fitness forces of selection in an age-structured population
Source: Commun Biol. 2023 Sep 5;6:909. doi: 10.1038/s42003-023-05260-9 (PMC10480192; doi:10.1038/s42003-023-05260-9)
Supplement: Supplementary file 3 — Description of Additional Supplementary Files [file 42003_2023_5260_MOESM3_ESM.pdf]

Description of Additional Supplementary Files

File name: Supplementary R Code
Description: R Code to reproduce Figures 2 and 3 in the main manuscript.
